# Zebrafish Models of Human Skeletal Disorders: Embryo and Adult Swimming Together

**DOI:** 10.1155/2019/1253710

**Published:** 2019-11-20

**Authors:** Marta Carnovali, Giuseppe Banfi, Massimo Mariotti

**Affiliations:** ^1^Gruppo Ospedaliero San Donato Foundation, Milan, Italy; ^2^IRCCS Orthopedic Institute Galeazzi, Milan, Italy; ^3^Vita-Salute San Raffaele University, Milan, Italy; ^4^Department of Biomedical, Surgical and Dental Sciences, University of Milan, Milan, Italy

## Abstract

*Danio rerio* (zebrafish) is an elective model organism for the study of vertebrate development because of its high degree of homology with human genes and organs, including bone. Zebrafish embryos, because of the optical clarity, small size, and fast development, can be easily used in large-scale mutagenesis experiments to isolate mutants with developmental skeletal defects and in high-throughput screenings to find new chemical compounds for the ability to revert the pathological phenotype. On the other hand, the adult zebrafish represents another powerful resource for pathogenic and therapeutic studies about adult human bone diseases. In fish, some characteristics such as bone turnover, reparation, and remodeling of the adult bone tissue cannot be found at the embryonic stage. Several pathological models have been established in adult zebrafish such as bone injury models, osteoporosis, and genetic diseases such as osteogenesis imperfecta. Given the growing interest for metabolic diseases and their complications, adult zebrafish models of type 2 diabetes and obesity have been recently generated and analyzed for bone complications using scales as model system. Interestingly, an osteoporosis-like phenotype has been found to be associated with metabolic alterations suggesting that bone complications share the same mechanisms in humans and fish. Embryo and adult represent powerful resources in rapid development to study bone physiology and pathology from different points of view.

## 1. Introduction: The Bone Tissue from *Danio rerio* to *Homo sapiens*

*Danio rerio* (zebrafish) is an elective model organism for the study of vertebrate development. This is due to the unique characteristics of the embryo such as large clutches (up to 250 embryos/week), small size, rapid external development, and transparency of the larval body. Such advantages encourage the use of live imaging and powerful genetic tools based on mutagenesis. Moreover, automated systems have been coupled with zebrafish embryo to create one of the most important *in vivo* methods for drug screening, drug discovery, and toxicity testing. The combination of these characteristics makes zebrafish an excellent animal model for developmental studies, basic biomedical research, drug development, and translational medicine studies [1].

Many structures and functions are common in the vertebrates, from human to fish. Bone is a heterogeneous tissue composed by a mineral phase, hydroxyapatite, organic phase (type I collagen, other structural proteins, and lipids), and water.

Zebrafish embryo is a powerful model to study osteogenesis, since five days after the fertilization of eggs the first mineralized vertebrae are already present and they can be visualized using vital dyes specific for the mineralized matrix [2]. The similarity of the adult skeletal structure between *Danio rerio* and *Homo sapiens* has hired zebrafish as animal model to study different aspects of skeletal physiology and pathology: bone metabolism, tissue turnover, and resorbing activity [3]. Bone tissue is not a simply protective and static scaffold for the adult organism, but it is a dynamic organ that stores crucial nutrients, proteins, minerals, and lipids and that is constantly remodeled [4, 5]. In addition, in recent years has emerged the endocrine role of the skeletal tissue because of its implication in the hormonal network, the energy metabolism, and the physiological regulation of several organs such as kidney, bone marrow, and muscles. [6].

In the last decade, several examples have been produced by the scientific literature concerning the introduction of zebrafish as model to study human bone diseases. In this review, we focused the attention on two different points of view in zebrafish skeletal studies: embryos and adult animals. Which resources and methods they offer to the medical research?

## 2. Zebrafish Embryo: Skeletal Development Studies and Screenings

### 2.1. Introduction

The main characteristics of the embryo such as rapid external development and transparency of the body make this model elective in organogenesis studies. The use of live imaging for mineralized tissue guarantees a complete representation of larval skeleton. Moreover, large clutches, small size, and automated systems have contributed to the creation of one of the most important *in vivo* methods for mutagenesis studies and drug discovery. The combination of such characteristics makes zebrafish an excellent animal model to search and study genes involved in skeletal development. When mutated, those genes can generate hard tissue dysfunctions similar to human diseases. Zebrafish embryo mutants for bone development are very helpful to perform drug development and translational medicine studies [7].

### 2.2. Morphants, Stable Knockdown, and Mutants

In the last years, morpholinos have become the elective techniques to quickly investigate the role of a specific gene in a particular developmental pathway, including skeletal system [8]. Several candidate genes responsible for bone alterations can be tested in zebrafish embryo using morpholino transient knockdown resource. Interestingly, a lot of morpholino-derived knockdown embryos (named morphants) actually exhibit a bone-related phenotype [8].

Recently, two alternatives for generating genomic mutations have been recently implemented in zebrafish such as transcription activator-like effector nucleases (TALEN) and the Crispr/Cas9 system [9].

Two large-scale “big genetic screens” searching for zebrafish embryo developmental mutants were carried out in Boston and Tubingen in the early 1990s. By a forward genetic approach, it is possible to understand the role of the mutated gene in normal development. From the big screens, more than 50 embryo mutants with defects in cartilage and bone development were identified [10]. Among these, several mutants have shown high correspondence of clinical output with human patients affected by skeletal diseases. The high similarity between human and fish about morphology and function of the bone tissues suggests that mutations in orthologue genes give rise to a similar effect in humans as well as in fish.

Osteogenesis imperfecta, scoliosis, arterial calcification, and osteoarthritis are some examples of human bone disorders modeled in zebrafish embryos derived from the genetic screenings [11].

#### 2.2.1. Relationship between Osteopenia and Anemia

Iron overload is a risk factor for osteoporosis but the correlation between iron deficiency and bone metabolism is not clear. In zebrafish, the *weissherbst* (weh (tp85c)) mutant (defective in ferroportin 1 gene, *fpn1*) exhibited a defective iron transport with severe hypochromic anemia. After the analysis of bone and cartilage during development, weh (tp85c) mutant larvae exhibit defects in the vertebral mineralization and osteoblast-specific gene expression. These data suggest that iron deficiency anemia affects bone formation modulating BMP signaling pathway in zebrafish embryos [12].

#### 2.2.2. Generalized Arterial Calcification of Infancy

Recently, generalized arterial calcification of infancy (GACI) has been linked to ectonucleotide pyrophosphatase/phosphodiesterase 1 (ENPP1) together with some cases of pseudoxanthoma elasticum (PXE). The mutation in the gene *enpp1* induces in human patients ectopic mineralization of soft tissues and arteries, increasing the risk of arteriosclerosis and cardiovascular diseases. Zebrafish *enpp1* mutants exhibit as well ectopic calcifications in several soft tissues suggesting a similar role of the protein in the calcium metabolism [13]. The conservation of the ENPP1 pathway will help to understand the mechanisms associated with pathological calcification processes in zebrafish as well as in humans.

#### 2.2.3. Collagenopathies and Osteogenesis Imperfecta

The type I collagenopathies are a group of connective tissue disorders caused by mutations in type I collagen gene. Various forms of osteogenesis imperfecta (OI) and the Ehlers–Danlos syndrome (EDS) are human bone diseases derived from these defects. Several zebrafish mutants in collagen genes have been used to dissect the bone structural regulation system and pathogenesis of bone fragility [14].

Osteogenesis imperfecta (OI) is a heterogeneous group of diseases characterized by susceptibility to bone fractures. Most patients are defective in the pathway of type I collagen biosynthesis and tissue organization with autosomal-dominant or autosomal-recessive inheritance. Most cases of OI are inherited, although some cases are the result of *de novo* mutations. The severity of the disease phenotype depends on the specific genetic defect [15]. Recently, a whole-exome sequencing in Korean OI patients identified two novel variants in the *bone morphogenetic protein* 1 (*bmp1*) gene (c.808A>G and c.1297G>T). Both variants generate a hypofunctional protein BMP1 as demonstrated using zebrafish embryo knockdown and rescue experiments. Indeed, mutant BMP1 cDNAs did not rescue in the *bmp1a* mutant the fin ruffling, which is the phenotype due to a reduced C-propeptide cleavage and consequent defective assembly of collagen helix [16].

Genes involved in the endoplasmic reticulum (ER) secretion pathways have been tested with success in zebrafish embryo as candidates for bone fragility in OI pathogenesis such as membrane bound transcription factor peptidase site 1 (MBTPS1) [17] and plastin-3 (PLS3) [18].

In addition, Sec24d-deficient zebrafish mutant *bulldog* exhibited defects in protein export system from the cellular ER generating a syndromic form of osteogenesis imperfecta [19]. The importance of ER function in OI pathogenesis has been evidenced in *Chihuahua* mutant embryos that carry a G574D (p. G736D) substitution in the α1 chain of type I collagen, where the administration of chemical chaperones 4PBA and TUDCA ameliorated bone mineralization in larvae [20].

#### 2.2.4. Scoliosis

Idiopathic scoliosis is a form of congenital vertebral malformation affecting 2-3% of children in the world, and it is characterized by a curvature of the spine in the absence of structural defects of the vertebral body. The pathogenic mechanisms of idiopathic scoliosis remain poorly understood but new and more informative animal models of scoliosis may contribute to the clarification of the molecular basis of the disease. It has been demonstrated that morphological defects of the zebrafish notochord can produce congenital vertebral defects in the latter stages. Three different recessive mutations on collagen type VIII alpha1a (col8a1a) gene were found in zebrafish leviathan mutant. The reduction of the collagen production affects the normal development of the embryonic notochord and generates vertebral column malformations at the adult stage [21].

Recently, a forward mutagenesis screening has been performed in zebrafish to identify new genetic targets for idiopathic scoliosis. The zebrafish recessive mutant *scoliosis* shows a spinal curvature without vertebral malformations. In *scoliosis*, the genome sequencing identified a nonsense mutation in *kinesin family member 6* (*kif6* (gw326)) gene [22]. A transgenic zebrafish for mutated-*kif6* gene developed a scoliosis phenotype identical to *scoliosis* mutant, confirming that spine curvature is due to the loss of KIF6 function. Overall, these findings demonstrate the power of the zebrafish screening resource to identify new candidate genes for human idiopathic scoliosis.

Still talking about mutants, zygotic protein tyrosine kinase 7 (Z*ptk7*) mutant zebrafish develops scoliosis-like phenotype at late larval and early juvenile stage associated with spinal curvatures of variable severity and normal vertebral bodies. PTK7 is a receptor tyrosine kinase conserved from human to fish and implicated in Wnt, Semaphorin/Plexin, and vascular endothelial growth factor (VEGF) signal transduction. Interestingly, maternal-zygotic ptk7 (MZptk7) mutant embryos, characterized by complete loss of function, show the scoliosis-like phenotype very early during spine development underlining the importance of the maternal PTK7 in the initial stages of skeletal development. After this study, a novel mutation that disrupts PTK7 function has been discovered within a single scoliotic patient, consistent with zebrafish data about the dysregulation of Wnt pathway in disease pathogenesis [23].

Recently, several genome-wide analyses have been made to identify new genes involved in the scoliosis. Among them, one has identified loci on chromosomes 10q24.31 and 6q24.1 associated with susceptibility of idiopathic scoliosis in the Japanese, Chinese, and European ancestry populations. From this study, two genes were found to be significantly associated with the disease: G protein-coupled receptor 126 (GPR126) and ladybird homeobox 1 (LBX1) [24]. GPR126 is a member of the adhesion GPCR family and is highly expressed in the human cartilage and in the chondrocytes of the vertebral body of the mouse embryo. Transient knockdown created in zebrafish embryo by the injection of gene-specific antisense oligonucleotide (morpholino) demonstrated that GPR126 is involved in the mineralization of the developing spine [25].

The role in axial bone development of ubiquitously expressed human LBX1 was studied using both gain-of-function and loss-of-function approaches in zebrafish. Overexpression of human or zebrafish lbx1 gene in zebrafish embryo generates axial developmental defects including defects in body curvature which, in some cases, are conserved up to the juvenile stage [26].

Another genome-wide association study on independent female cohorts from the United States of America and Japan identified an enhancer region distal to *paired box* 1 (*Pax1*), a gene originally associated with spinal development and emerged from studies on undulated mouse strain carrying a missense mutation in Pax1 [27]. Such region, named *Xe1*, together with another candidate, PEC7, has been tested with green fluorescent protein-Tol2 (GFP-Tol2) enhancer assays in zebrafish embryos. Both regions displayed functional enhancer activity in the developing spine, specifically corresponding to somitic muscles [28].

Another extended genome-wide association study using independent Japanese and Chinese populations identified a susceptibility locus for idiopathic scoliosis on chromosome 9p22.2. The crucial polymorphisms were found in the intron 3 of *basonuclin-2* gene encoding one of the most evolutionarily conserved zinc finger transcription factors (BNC2), with 98% of protein homology between mouse and human. BNC2 is expressed in many human tissues, including epithelial and germ cells, and its overexpression produced spine curvature in developing zebrafish in a dose-dependent manner [29].

#### 2.2.5. Raine Syndrome

Raine syndrome (RNS) is an autosomal recessive genetic disease associated with generalized osteosclerosis, intracerebral calcifications, and facial dysmorphism. *Family with sequence similarity 20 member C* (*fam20c*) is a gene encoding a secreted protein kinase (FAM20C), which binds calcium and phosphorylates substrates involved in bone mineralization processes like osteopontin [30]. Raine syndrome was found to be associated with biallelic mutations in *fam20c*, located on human chromosome 7p22.3 [31]. Zebrafish homozygous mutant in the gene *fam20b* revealed that Fam20b has a role in cartilage matrix production and skeletal development, showing similar morphological alterations found in human RNS patients [32].

#### 2.2.6. Craniosynostosis

Craniosynostosis is a developmental defect of the skull with premature fusion of the cranial sutures [33]. In humans, mutations in the gene coding for cytochrome P450, family 26, subfamily B, and polypeptide 1 (CYP26B1) are associated with radiohumeral fusions and other skeletal dysfunctions including craniosynostosis [34]. *CYP26B1* encodes a member of the cytochrome P450 superfamily which is upregulated during osteoblast differentiation and responsible for retinoic acid degradation, suggesting that a reduction in retinoic acid signaling is required for normal osteogenesis. In humans, clinical cases of craniosynostosis have shown important phenotypic similarities with two zebrafish cyp26b1 mutants, named dolphin (dol) and stocksteif (sst), in which can be observed deficiencies in cartilaginous structures, bone fusions, and hypermineralization in facial and axial bones [34].

In Saethre-Chotzen syndrome, mutations in *transcription factor* 12 (*TCF12*) or *twist-related protein* 1 (*TWIST1*) genes ablate the coronal suture, as observed in human and rodent studies. Interestingly, TALEN-mediated inactivation of homolog genes in zebrafish embryos generates specific loss of the coronal suture [35].

Several genetic studies with next-generation sequencing confirmed that a genetic cause of craniosynostosis can be identified in one-quarter of cases. Among these, mutations in two genes, TCF12 and ERF, were found to be predominant, whereas other mutations in CDC45, SMO, and SMAD6 are suggested to be involved in the pathogenesis [36]. These data support the importance of the sequencing approach as a diagnostic method.

#### 2.2.7. Lenz–Majewski Syndrome

Lenz–Majewski syndrome (LMS) is a genetic disease characterized by mental retardation and generalized craniotubular hyperostosis. By using whole-exome sequencing, different missense mutations have been identified in the gene *ptdss1*, which encodes phosphatidylserine synthase 1 (PSS1). The expression of the mutant *PTDSS1* RNA in zebrafish embryo caused several dose-dependent developmental defects in 6% to 40% of embryos. Craniofacial anomalies, trunk angulation, small or absent eyes, and abnormal cartilage were observed in the affected embryos [37].

Recently, a transpose-mediated transgenesis has been used to stably express wild-type and mutant forms of human PTDSS1 ubiquitously or specifically in chondrocytes, osteoblasts, or osteoclasts in zebrafish embryo to better evidence the bone phenotype [38].

#### 2.2.8. Auriculocondylar Syndrome

Auriculocondylar syndrome (ACS) is a human birth defect syndrome showing alteration in ear and mandible development. It has been hypothesized that defects in signaling molecules associated with the endothelin signaling pathway could affect the ability of the neural crest cells to form most of the bone, cartilage, and connective tissue of the face. In fact, gene disruption experiments of *endothelin-1* (*edn1*) in both mouse and zebrafish resulted in loss of identity of neural crest cells of the first pharyngeal arch with consequent misdifferentiation of lower jaw structures into maxillary-like structures [39]. Subsequently, to better clarify the genetic basis, other regulators of endothelin pathway have been knockdown in zebrafish embryos such as Nkx2.5, resulted in defects in both ventral and dorsal pharyngeal arch [40], or Wdr68 which is required for edn1 expression and formation of the ventral part of Meckel's cartilage [41].

#### 2.2.9. Pseudoxanthoma Elasticum

Pseudoxanthoma elasticum (PXE) is a mineralization disease related to a more severe vascular calcification syndrome called generalized arterial calcification of infancy (GACI). PXE is associated with mutations in the *ATP-binding cassette subfamily C member 6* (abcc6) gene. ABCC6 is a membrane transporter involved in the ATP/AMP/pyrophosphate pathway. In fact, pyrophosphate regulates deposition of calcium and other minerals in the tissues. Patients with PXE show reduced levels of circulating vitamin K and calcified lesions in the skin, eyes, and blood vessels [42]. The mutation in the zebrafish orthologue *abcc6a* results in extensive hypermineralization of the axial skeleton [11]. Vitamin K administration to zebrafish embryos rescued the pathological phenotype restoring normal levels of mineralization [43] underlining the importance and usefulness of zebrafish in rapid gene-knockdown and drug screenings.

#### 2.2.10. Osteoarthritis

Several studies on human osteoarthritis (OA) have been addressed to the genetic component of the disease [44, 45]. In this context, zebrafish represents a powerful model to study the role of candidate genes in AO pathogenesis.

Histological analysis of the cartilage of OA patients indicated that the tissues possess the characteristics of an endochondral ossification process. For that reason, the best candidate genes for OA were those related to the endochondral ossification. In situ hybridization and transgenic reporter experiments have been made with some of zebrafish homologous genes: Mcf2l, Gdf5, PthrP/Pthlh, Col9a2, and Col10a1. The spatiotemporal expression revealed that the genes were dynamically expressed during zebrafish skeletal development whereas some of them (col2a1 and col10a1) were specifically associated with chondrocyte hypertrophy *in vivo* [46].

More recently, it has been demonstrated, in zebrafish col11a2 mutants, that the protein is correctly produced but early degraded in forming joint cartilage. These changes in shape and structural properties affect joint function [47].

Zebrafish studies contributed also to the analysis of the role of inflammation in the pathogenesis of OA. In particular, a hyperactive variant of the receptor interacting protein kinase 2 (*ripk2*) gene, RIPK2^104Asp^, has been expressed in zebrafish embryos resulting in the upregulation of OA-associated genes [48]. In fact, RIPK2 is able to activate the innate immune response and the NF-*κ*B pathway enhancing the inflammatory response, which increases the risk of OA.

All these studies demonstrated the utility of zebrafish embryo model in functional studies of genes playing a role in OA pathogenesis.

#### 2.2.11. Cohesinopathies

Roberts syndrome (RBS) and Cornelia de Lange syndrome (CdLS) are rare genetic diseases with overlapping phenotype and associated, among others, with craniofacial and limb abnormalities. Mutations were found in genes coding for the establishment of sister chromatid cohesion *n*-acetyltransferase 2 (ESCO2), SMC3, SCC2/NIPBL, SMC1, RAD21/MCD1, and proteins involved in the cell-cell cross-talk and related to the cohesin family [49].

The zebrafish regenerating fin model has been used to test the role of ESCO2 in skeletal development and disease. The gene *esco2* was found upregulated during fin regeneration, and the knockout embryo was defective for bone growth in regenerating fins, confirming the role of *esco2* in skeletal morphogenesis and in the pathogenesis of cohesinopathies [50].

#### 2.2.12. Gaucher Disease

Gaucher disease (GD) is a genetic disease caused by loss of lysosomal glucocerebrosidase (GBA1), responsible for multiorgan malformations and skeletal defects. Impairment of osteoblastic activity and enhanced macrophage-dependent bone resorption have been hypothesized as pathogenic mechanisms [51]. In order to clarify the molecular basis of the GD, a transient *Gba1* knockout has been produced in zebrafish embryo using morpholino techniques. The phenotype resulted in an increased reactive oxygen species production and defective canonical Wnt signaling which caused impaired osteoblast differentiation and reduced osteogenesis [52].

### 2.3. Dysbiosis

There is growing scientific literature affirming that a disequilibrium of the gut microbiota (dysbiosis) is associated with several human diseases such as allergy, asthma, metabolic, cardiovascular, and bone diseases [53]. Different effects of the intestinal flora on immune cells, hormones, fatty acids, neurotransmitters, and vitamins can mediate the interaction between microbiome and skeletal system [54].

Some studies about the effects of microbiota on zebrafish bone metabolism have been recently presented. In particular, *Lactobacillus rhamnosus*, a component of the human gut flora, has been administered chronically with the diet to zebrafish embryo. Interestingly, backbone centra of the treated larvae exhibited more extensive calcification than the untreated controls. In fact, *L. rhamnosus* stimulates the expression of genes involved in osteogenesis such as runt-related transcription factor 2 (runx2), Sp7 transcription factor (sp7), matrix Gla protein (mgp), and bone gamma-carboxyglutamate (gla) protein (bglap) and inhibits the expression of sclerostin (sost), a bone formation inhibitor [55].

### 2.4. High-Throughput Screening Resource

The zebrafish has become an elective model for the phenotype-based drug discovery. Similarities and differences between human and zebrafish biology drove the use of zebrafish as a resource for *in vivo* screenings. High-throughput automatized technologies and zebrafish embryo can generate, together, a powerful resource for drug screening, target identification, pharmacology, and toxicology [56].

In the past, high-throughput *in vivo* screening using larval zebrafish has been developed to facilitate the identification of compounds with effects on osteogenesis. In these experiments, vitamin D3 analogs (VD) and intermittent administration of parathyroid hormone (PTH) have been used as positive control molecules. The live staining of mineralized tissues during skeletal development revealed that VD and PTH possess the same anabolic effect highlighted in humans. Such result underlined the metabolic similarity of the bone tissue between humans and fish and proposes *in vivo* screening as a powerful method to isolate new anabolic compound for human bone-loss diseases [57].

Recently, an automatized high-throughput optical projection tomography screening has been applied to developing zebrafish embryos to test several classes of teratogens for their effects on cartilage formation [58]. Tomographic technique is particularly suited for quickly analyzing and recording any changes in skeletal pattern during the drug screening experiment.

Zebrafish embryo models recapitulating human bone diseases (e.g., idiopathic scoliosis mutant) can be used to screen for a compound able to rescue the pathological phenotype. This method facilitates the identification of novel compounds useful for further drug development.

After hepatotoxicity, immunotoxicity, neurotoxicity, and/or reproductive toxicity studies, environmental toxicology has hired zebrafish embryo to screen pollutants affecting skeletal tissues (osteotoxicity) by acute or chronic exposure to different environmental insults [59].

### 2.5. Transgenic Embryos and Bone Imaging

The transparency of the zebrafish larva leads itself to the use of noninvasive staining methods which include vital dyes and transgenic fluorescent animals. Live staining of embryos with fluorescent dye such as calcein [60] and alizarin red S [61] has been developed to visualize calcified skeletal structures. Transgenic fish are very useful when fluorescent proteins are expressed in skeletal tissues. In the last years, different transgenic reporter lines have been produced to mark cartilage or bone [62]. The cartilage-specific expression of the reporters in Tg (*Col2a1aBAC:mCherry*)*hu5900* [63] and Tg (*1.7col2a1a:mCherry-caax*) [64] permits us to highlight tissue malformation in the presence of chemical treatment or to diagnose specific diseases like osteoarthritis.

Reporter lines using *osterix*/*sp7* promoter as Tg (*sp7:EGFP*)*b1212* [65], *medaka* osx-mCherry [66], and Tg (*Ola. Sp7:NLS-GFP*)*zf132* [67] are useful to study the timing of the osteoblast differentiation, which is involved in craniofacial congenital malformations [68].

Reporters using cathepsin K promoter as *CTSK-DsRed* [69] or *Ctsk YFP* [70] have been developed to observe the differentiation of osteoclasts and their role in the normal development of different organs as well as in pathological conditions. In particular, *Tg* (ctsk*:mEGFP*) transgenic line has been used in a multitransgenic approach together with heat-shock-induced receptor activator of nuclear factor kappa-B ligand (RankL) expression. The RankL-mediated osteoclast differentiation causes an osteoporotic phenotype in medaka embryo [71].

Other transgenic lines have also been applied for joint studies. For example, trps1j1271aGt expresses GFP in joint regions of wild-type fish and it has been used to elucidate the regulatory pathways of differentiation and organization of joint cells in physiological and pathological conditions [72].

## 3. Adult Zebrafish

### 3.1. Introduction

In fish, some characteristics such as bone turnover, repair, and remodeling of the adult bone tissue cannot be found in embryonic or juvenile stages. In addition, adult fins and scales represent unique anatomical features with undoubted advantages like transparency and the presence of mineralized tissue similar to human lamellar bone. The same dyes used for live embryo bone tissue staining can be used in adult fish, on both live fish and fixed sample to highlight the scale mineral matrix [73] or caudal fin rays [74]. For these reasons, the adult zebrafish represents an innovative and readily available resource for studying the regulatory mechanisms of adult bone metabolism at cellular and molecular levels as well as the pathogenesis of bone diseases or bone complications of human diseases. Several examples of bone disease models in adult zebrafish have been generated in the last years.

### 3.2. Mutant Zebrafish in Adult Stage and Bone Disease Models

The identification of zebrafish mutants that recapitulate human mineralized craniofacial, dental, and skeletal system disorders can be used at adult stage as models to study the progression and the pathological effects (primary or secondary).

Years ago, zebrafish mutant *Chihuahua* was isolated in a forward-genetics screen of adult fish searching for skeletal abnormality using X-ray [75]. Heterozygous fish was characterized by bone fragility, altered vertebral shape, and frequent rib fractures [76]. The same clinical evidences can be found in human patients affected by heterozygous OI. Thus, the adult zebrafish mutant *Chihuahua* can be considered as a model of human osteogenesis imperfecta useful to elaborate new therapeutic strategies [20].

Few years ago, a large-scale forward genetic chemical mutagenesis screening led to the identification of 7 adult homozygous recessive mutants with skeletal disorders including craniofacial defects, suture fusion, morphological or numerical alteration of skeletal elements, and scoliosis [77].

The regulatory mechanisms of postembryonic development are still poorly understood. Recently, a screening for phenotypes affecting the adult zebrafish has been performed with the identification of 72 adult viable mutants showing defects in skeleton and pigmentation [78].

These mutant models can be used in the adult stage to study long-term complications of bone diseases as well as the pathogenesis of defects in postembryonic development.

### 3.3. Transgenic Models of Bone Disease in Adult Zebrafish

Fibrodysplasia ossificans progressiva (FOP) is a rare human skeletal disease caused by constitutively activating mutations in the gene ACVR1. Heterotopic ossification (HO) throughout skeletal muscle, tendons, and ligaments has been reported in human patients with generation of bone malformations, vertebral fusions, and osteochondromas [79].

Since the embryonic overexpression of constitutively active Acvr1 in zebrafish results in embryonic lethality, heat-shock inducible expression constructs for acvr1Q204D have been used to create a conditional mutant model of FOP in adult zebrafish [80].

### 3.4. Microgravity-Induced Osteoporosis

Progressive bone loss is a serious side effect of the long-term permanence in the absence of gravity. Clinical studies on astronauts revealed that an average of 1 to 2 percent of bone mass can be lost each month [81]. Several data have been produced examining the effects of simulated microgravity on different animal models. In particular, the skeleton of adult fish exposed to simulated microgravity resulted in growth alteration, reduced ossification, and distortion of some skeletal elements [82, 83].

### 3.5. Mechanical Loading

It is known that physical exercise modulates bone mass in all vertebrates; nevertheless, the mechanisms are still largely unknown. We can suggest that the mechanical stress generates a musculoskeletal adaptation stimulating sensor systems localized on bone cells [84, 85].

Adult zebrafish represents an innovative model to study the effect of the mechanical loading on bone mass and metabolism, in absence of gravity charge.

Recently, an increased physical exercise has been applied to adult zebrafish for four weeks using a specific swim tunnel. The results demonstrated that the exercise stimulated increases in bone-forming osteoblasts, bone volume, and mineralization [86].

About cartilage tissue, it has been demonstrated that chondrocytes possess a high-rate turnover in zebrafish adult spine and that age but not short-term intensive exercise can modulate spinal BMD [87]. The zebrafish vertebral cartilage represents a good model to study the homeostasis of adult articular cartilage in physiological and pathological conditions.

The knowledge of the molecular mechanisms of mechanical adaptation in adult bone can improve the development of new strategies of prevention and treatment in human diseases like osteoporosis.

### 3.6. Injury Models and Bone Regeneration Studies in Adult Zebrafish

#### 3.6.1. Fin Cut and Regeneration

Zebrafish is able to fully regenerate fins via dedifferentiation, proliferation, and redifferentiation of bone-forming cells. The growth of new skeletal structures is coordinated with other tissues (blood vessels, epithelium, connective tissue, immune cells, and nerves) in a complex regulatory mechanism which is still to be understood [88]. These studies will contribute to the elucidation of cellular and molecular pathways involved in cross-talk between bone cells and the surrounding tissues in adult vertebrates.

Adult fin regeneration can be analyzed also in pathological models like type 1 diabetes as bone complication of metabolic disease. An impairment of caudal fin regeneration has been observed in hyperglycemic zebrafish established by streptozotocin injection [89].

#### 3.6.2. Skull Trepanation

The skull of adult zebrafish can be also used as a model for regeneration studies through the trepanation of calvarial bones. The hole in the bone tissue is repaired by a metabolic reactivation of surrounding osteoblasts [90]. These data suggested that intramembranous regeneration (observed in skull and fin) is characterized by the dedifferentiation of osteoblasts, proliferation, and redifferentiation into new osteoblasts [91].

#### 3.6.3. Jawbone Regeneration

It is known that several zebrafish cranial bones are formed by endochondral ossification. To elucidate the mechanisms of repair in cartilage-derived bones as well as their origins in the periosteum, a large-scale regeneration model has been established in adult jawbone. After resection of zebrafish jaw, a mixed cartilage/bone cell type emerges from bone-lining cells and starts to produce cartilage matrix early and mineralized matrix later [92].

Chondroid bone repair processes can also coexist with intramembranous ones during bone healing. This model is elective to study *in vivo* the osteochondral regulation during cell specification and function.

#### 3.6.4. Scale Regeneration

The adult zebrafish scales are also able to regenerate rapidly after loss. Recent data indicated that osteoblast-precursor cells differentiate and proliferate establishing a primordium of the new scale. In posterior scale regions, mineralized grooves (radii) are radially formed during the deposition of new rings [93]. This model is elective to study *in vivo* the direct differentiation processes from adult stem cell-like precursors to bone-forming cells. The presence of osteoclasts in the scale matrix and the transparency of the anatomical structure encourage the use of this model to investigate the role of catabolic phenomena in bone tissue regeneration [94, 95].

#### 3.6.5. Long-Bone Fracture on Caudal Fin

Since zebrafish caudal fin possesses unique characteristics of accessibility and transparency, it has been introduced to investigate the reparative capacity of adult bone tissue.

A caudal fin ray crush injury has been used to develop a model of vertebrate long-bone fracture, which easily analyzes mineralized matrix deposition and time-regulated gene expression can be easily analyzed during the repair process [96]. The bone callus formation has been recently analyzed in various pathological conditions. In particular, it has been found to be delayed during fracture repair in OI adult model and in *csf1ra* mutant, which has reduced osteoclast numbers, due to a reduced remodeling function [97]. In addition, the role of immune cells in bone repair in the presence of *Staphylococcus aureus* has been investigated showing that when fractures are infected, neutrophils are retained and delayed repair due to a prolonged inflammatory state [97].

The regeneration program, typical of the extensive fin injury, should not be activated for a single ray fracture but rather a tissue repair process, much more similar to a wound healing. The identification of the key processes that regulate the tissue response after bone fracture may contribute to the improvement of bone repair in humans.

### 3.7. Osteoporosis in Axial Skeleton

Osteoporosis is a bone disease in which mineralized tissue becomes weak and the risk of fracture increases significantly, especially in the spine. Zebrafish spine, in addition to being anatomically and histologically similar, is also axially loaded like humans and shows similar pathologies to humans during ageing [98].

An osteoporotic phenotype is induced in the spine by overexpression of RankL and GFP in transgenic medaka lines with heat-shock-controlled osteoclastic promoter. When RankL is induced in adult stage, osteoclasts migrate from the intervertebral regions to the centra of the vertebral bodies causing severe degradation of the mineralized matrix [71].

It is known that an accumulation of elevated iron in the body is an independent risk factor for osteoporosis. Adult zebrafish has been used to model an osteoporosis phenotype using high iron stress (FAC, ferric ammonium citrate). Iron overload decreases bone mineralization in axial skeleton of adult zebrafish. In addition, osteoblast-specific gene expressions were found significantly modulated after iron exposure [99].

### 3.8. Osteoporosis in Dermal Skeleton

Adult zebrafish scale has been recently introduced as read-out system for bone metabolism studies [100, 101]. The transparency and the size of the scale facilitate manipulation, treatment, and observation of the results under a stereomicroscope. In addition, several parameters such as biochemical and cellular markers, bone matrix architecture, bone deposition, bone resorption, and behavior of bone cells (scleroblasts and osteoclasts) can be easily analyzed in the scale [102, 103]. These advantages make scale an ideal sample to study mineralization and remodeling mechanisms in the pathological models.

Recently, secondary osteoporosis has been reproduced in adult zebrafish after treatment with prednisolone [104], a glucocorticoid known to have the same effects in humans. The osteoclast-dependent resorbing activity has been observed in explanted scales from treated fish using the specific staining for mineralized matrix and histological staining to highlight osteoclast activity. The treatment with alendronate, an antiosteoporotic drug, has surprisingly resulted in significant protective effects in prednisolone-treated fish [73]. The scale can be also explanted and cultured in microplates to screen for chemical compounds with antiosteoporotic properties [100]. These data sustain the utility of the scale as “read-out” system to study human bone metabolism and found new pharmacological therapy [105].

### 3.9. Obesity, Diabetes, and Bone Complications

Zebrafish has been recently introduced to model chronic diseases of metabolism such as obesity and type 2 diabetes and, in particular, to identify genetic variants associated with these conditions in humans [106].

In fact, zebrafish represents a powerful animal model to study the metabolic diseases and their complications [107]; nevertheless, few data are available about the relationship between glucose metabolism, fat, and bone homeostasis.

#### 3.9.1. Blood Glucose and Bone Complications

It is known that the skeletal metabolism is affected in diabetes patients [108] but the molecular mechanisms are still unclear.

To clarify the correlation between blood glucose level and bone metabolism, a chronic hyperglycemia was established in a new zebrafish model of type 2 diabetes by glucose administration in the water [109]. The increase of basal glycaemia induces alterations in size and structure of the retinal blood vessels resembling to the human diabetic retinopathy. The scales of glucose-treated fish fail to depose new mineralized matrix and show osteoclastic-dependent bone resorption. In fact, hyperglycemic fish scales are associated with a significant decrease of alkaline phosphatase activity and increase of tartrate-resistant acid phosphatase activity, together with alterations in other bone-specific markers [109]. These data indicate that the imbalance of blood glucose affects bone metabolism, which leads to the osteoporotic-like phenotype in the fish scale. The bone loss is also a clinical complication in diabetic human patients. The zebrafish model of hyperglycemia-derived bone loss can contribute to the elucidation of *in vivo* the molecular mechanisms of bone complications as demonstrated by the analysis of advanced glycation end-products (AGEs) and PTH levels in the fish blood [110]. A hyperglycemic adult zebrafish can also be used as a screening model for new therapeutic strategies against long-term complications.

Recently, zebrafish models of diabetes have been used to test glucose lowering natural compounds [111]. One of these, the liquiritigenin (LTG), a flavonoid extracted from *Glycyrrhiza glabra* roots, significantly prevents the onset of the hyperglycemia in adult zebrafish and systemic alterations such as increase of AGEs and PTH levels in the blood [110]. Interestingly, LTG inhibits the glucose-dependent increase of osteoclastic activity and relative bone-loss phenotype in zebrafish scales. Gene expression analysis shows that LTG reequilibrates bone metabolism preventing the alteration of crucial bone regulatory genes [110].

This study confirmed that hyperglycemic zebrafish is a powerful tool to screen new compounds active on blood glucose level and bone complications.

#### 3.9.2. Visceral Fat and Bone Complications

It is known that the risk and the number of some fractures (proximal humerus, femur, and ankle) are higher in obese people. Bone mineral index (BMI) and bone mineral density (BMD) are correlated and the mechanisms of this association *in vivo* are linked to metabolic impairment such as adipokines (leptin) overproduction and higher aromatase activity [112].

Since lipid metabolism and the adipogenic mechanisms are conserved between fish and mammals, zebrafish has been developed as an important model system to study fatty acid metabolic syndrome [113] and obesity [114]. Nevertheless, no data have been produced about the correlation between obesity and bone metabolism in adult zebrafish.

Recently, an obese adult zebrafish has been created by high-fat diet administration to study the metabolic relationship between fat and bone [115]. Visceral fat was found clearly accumulated in treated fish versus untreated ones. In addition, metabolic alterations similar to human obese patients have been evidenced by fish blood analysis. Glycaemia and the levels of insulin, adiponectin, and leptin were found to be altered in high-fat diet fish. In this condition, the elevated glucose level stimulates the generation of AGEs in the fish blood, which are responsible for diabetic complications as retinopathy and osteoporosis.

In fact, the decrease in adiponectin and increase in leptin, which are common features in human and other obesity animal models, are associated with bone catabolic processes [116, 117].

In obese fish, osteoporosis has been found in scales as large bone resorption lacunae associated with an intense TRAP activity, whereas ALP was found decreased. The gene expression analysis supports the hypothesis that the unbalance of the RANL/RANK/OPG pathway in obese fish alters bone metabolism in favor of osteoclastic activation [115].

The study of adult obese zebrafish suggests that fat accumulation leads to an alteration of glucose metabolism (insulin-resistance) with generation of AGEs and unbalanced production of important adipokines such as adiponectin and leptin. These metabolic changes synergize to alter bone metabolism in zebrafish scales inducing an osteoporotic-like phenotype. The zebrafish obesity model can be used to elucidate *in vivo* the molecular mechanisms of metabolic alterations and bone complications in human obese patients.

### 3.10. Advanced Skeletal Imaging Resources for Adult Animals

Several imaging resources may be used to facilitate the investigations on the adult zebrafish models of bone disease contributing to the knowledge of the pathogenic mechanisms of human counterpart. A lot of imaging resources can be adapted for small animal models like rodents or fish.

Recently, Time-Gated Optical Projection Tomography (TGOPT) has been used in adult zebrafish to visualize in 3D the anatomical body structure without chemical contrast [118].

In addition, whole adult zebrafish can be scanned by microcomputed tomography (*μ*CT), permitting the analysis of the skeleton and the determination of BMI for each bone scan section. Total-body *μ*CT scans can be applied in pharmacological studies where the 3D reconstruction of the zebrafish bone system is used to detect alterations in bone structures after particular treatments like dietary supplementation [119].

Zebrafish has been used to assess age-related degenerative changes in the vertebral bone by using *μ*CT scanning. The microstructural analysis of the trabecular thickness, trabecular number, and star volume of the tissue space and trabeculae reported that the size of the trabecular bone was reduced with age in adult zebrafish [120].

*In vivo* high-resolution images on adult zebrafish skeleton can be also obtained with spectral-domain optical coherence tomography (SD-OCT). Recently, bone defects due to a prednisolone treatment have been quantitatively evaluated using the SD-OCT images at different time points during a period of 21 days [121].

X-ray radiography has been already introduced in adult zebrafish mutant screening to detect abnormalities in skeletal morphology [75].

## 4. Conclusions

*Danio rerio* (zebrafish) is an elective model organism for the study of vertebrate skeletal biology because of its high degree of homology between human and zebrafish in terms of structure, function, and regulation mechanisms. Zebrafish embryos, because of the optical clarity, small size, and fast development, can be easily used to (1) study osteogenesis evaluating differentiation, matrix deposition activity, and cross-talk of skeletal cells, (2) create and isolate mutants modeling human bone diseases, and (3) test in high-throughput screenings new chemical compounds for the ability to revert bone defects. On the other hand, the adult zebrafish represents a different and powerful resource useful for pathogenic and therapeutic studies about adult human bone diseases because some functions like bone turnover, repair, degeneration, and metabolic responses are not mature in embryos. For these reasons, the adult zebrafish represents an innovative and readily available resource for studying (1) the regulatory mechanisms of adult bone metabolism at cellular and molecular levels, (2) the pathogenesis of bone diseases, and (3) bone complications of different human diseases. Several examples of bone disease models in adult zebrafish have been generated in the last years.

Many disease models with bone alterations have been established in adult zebrafish such as long-bone fractures, osteoporosis, genetic disease as osteogenesis imperfecta, diabetes, and obesity.

In conclusion, zebrafish embryos and adults should swim together to allow us to understand bone physiology and pathology from all points of view.
